# Reactivation of the Neurogenic Niche in the Adult Zebrafish Statoacoustic Ganglion Following a Mechanical Lesion

**DOI:** 10.3389/fcell.2022.850624

**Published:** 2022-03-15

**Authors:** Simone Schwarzer, Devavrat Ravindra Rekhade, Anja Machate, Sandra Spieß, Michaela Geffarth, Diana Ezhkova, Stefan Hans

**Affiliations:** CRTD—Center for Regenerative Therapies Dresden, Center for Molecular and Cellular Bioengineering (CMCB), Technische Universität Dresden, Dresden, Germany

**Keywords:** PNS, inner ear, neuronal stem cells, regeneration, zebrafish

## Abstract

Sensorineural hearing loss is caused by the loss of sensory hair cells and/or their innervating neurons within the inner ear and affects millions of people worldwide. In mammals, including humans, the underlying cell types are only produced during fetal stages making loss of these cells and the resulting consequences irreversible. In contrast, zebrafish produce sensory hair cells throughout life and additionally possess the remarkable capacity to regenerate them upon lesion. Recently, we showed that also inner ear neurogenesis continues to take place in the zebrafish statoacoustic ganglion (SAG) well into adulthood. The neurogenic niche displays presumptive stem cells, proliferating Neurod-positive progenitors and a high level of neurogenesis at juvenile stages. It turns dormant at adult stages with only a few proliferating presumptive stem cells, no proliferating Neurod-positive progenitors, and very low levels of newborn neurons. Whether the neurogenic niche can be reactivated and whether SAG neurons can regenerate upon damage is unknown. To study the regenerative capacity of the SAG, we established a lesion paradigm using injections into the otic capsule of the right ear. Upon lesion, the number of apoptotic cells increased, and immune cells infiltrated the SAG of the lesioned side. Importantly, the Neurod-positive progenitor cells re-entered the cell cycle displaying a peak in proliferation at 8 days post lesion before they returned to homeostatic levels at 57 days post lesion. In parallel to reactive proliferation, we observed increased neurogenesis from the Neurod-positive progenitor pool. Reactive neurogenesis started at around 4 days post lesion peaking at 8 days post lesion before the neurogenesis rate decreased again to low homeostatic levels at 57 days post lesion. Additionally, administration of the thymidine analog BrdU and, thereby, labeling proliferating cells and their progeny revealed the generation of new sensory neurons within 19 days post lesion. Taken together, we show that the neurogenic niche of the adult zebrafish SAG can indeed be reactivated to re-enter the cell cycle and to increase neurogenesis upon lesion. Studying the underlying genes and pathways in zebrafish will allow comparative studies with mammalian species and might provide valuable insights into developing cures for auditory and vestibular neuropathies.

## Introduction

According to the World Health Organization, nearly 2.5 billion people will suffer from some degree of hearing loss in the next 2 decades, and at least one quarter will require hearing rehabilitation (https://www.who.int/news-room/fact-sheets/detail/deafness-and-hearing-loss). The vast majority of hearing impairment constitutes sensorineural hearing loss, which originates in the auditory part of the inner ear. The inner ear comprises six sensory patches containing mechanosensory hair cells that convert vestibular or auditory stimuli into electrochemical signals, as well as their innervating sensory neurons that transmit the signals to the brain *via* the eighth cranial nerve (Schwander et al., 2010). Whereas five sensory patches mediate vestibular function to perceive linear movement, gravity, and head rotation, hearing relies on a single sensory patch located in the cochlea. Damage to, or loss of, hair cells residing in the cochlea and/or their respective neurons results in sensorineural hearing loss. Unaddressed hearing loss can have severe impacts with respect to life quality and the socioeconomic status as people with untreated hearing loss are more likely to develop depression, anxieties, and feelings of inadequacy often reflected in lower household incomes (Tsakiropoulou et al., 2007). Moreover, recent epidemiological studies indicated that acquired hearing loss represents an independent risk factor for dementia and could account for approximately one-tenth of all cases (Livingston et al., 2017). Hence, unaddressed hearing loss amounts to significant economic burdens to individuals and society due to reduced productivity in the workplace as well as increased health care costs.

Despite the scale of the problem, treatment options are still largely limited, and no cell, molecular, or pharmacological therapies are currently available. To this aim, stem cell-based inner ear cell cultures derived from human embryonic stem cells as well as human-induced pluripotent stem cells provide a promising tool in the future. Stem cell-based inner ear cell cultures will be scalable and will allow generating transplantable sensory and neural progenitors *in vitro*, to replace the lost cell type *in vivo* (Chen et al., 2012; Koehler et al., 2017; Zine et al., 2021). In general, stem cell-based cell cultures use externally applied growth factors and small molecules to recapitulate the differentiation programs employed during development. In addition, direct reprogramming using transcription factor overexpression has been shown to be effective *in vitro* for the production of hair cell-like and neuron-like cells that display a transcriptomic profile resembling endogenous hair cells and neurons (Costa et al., 2015; Noda et al., 2018). However, irrespective of the methodology, robust and reproducible protocols are still missing due to the complex cellular and molecular nature of sensory and neural progenitor formation. Moreover, the cellular and molecular properties of transplantable cells to ensure functional restoration remain unclear.

A complementary approach relies on the analysis of *in vivo* models. Here, models either mimic the consequences of sensorineural hearing loss following the ablation of hair cells within the cochlea or neurons of the eighth cranial nerve or address the innate ability to replace lost cells upon damage in regeneration-competent organisms. Similar to humans, all mammalian models display no regenerative capacity. For instance, application of aminoglycosides, commonly prescribed antibiotics to treat bacterial infections, results in hair cell ablation in rats, mice, guinea pigs and Mongolian gerbils with no indication of subsequent regeneration (Astbury and Read, 1982; Forge, 1985; Taylor et al., 2008; Abbas and Rivolta, 2015). Similarly, application of the neurotoxin ouabain, an Na+/K+-ATPase inhibitor, results in the induction of cell death in most neurons of the eighth cranial nerve in mice, rats, and Mongolian gerbils with no signs of regeneration (Schmiedt et al., 2002; Lang et al., 2005; Fu et al., 2012; Wakizono et al., 2021). In contrast to mammals, all other vertebrates, such as birds, reptiles, amphibians, and fish have the remarkable intrinsic capacity to regenerate lost hair cells (Corwin and Cotanche, 1988; Ryals and Rubel, 1988; Stone and Rubel, 2000; Schuck and Smith, 2009). In particular, the zebrafish lateral line has emerged as a powerful model to study the events underlying hair cell regeneration in recent years due to its ease in applying aminoglycosides at larval stages, and single-cell RNA sequencing has recently revealed distinct stem cell populations driving this process (Stawicki et al., 2015; Lush et al., 2019). In sharp contrast, knowledge on the formation of inner ear neurons during regeneration in the regeneration-competent zebrafish is currently completely lacking. We recently showed the lifelong existence of a neurogenic niche in the eighth cranial nerve, also known as statoacoustic ganglion (SAG), in zebrafish (Schwarzer et al., 2020). We found that Neurod-positive progenitors proliferate and produce large amounts of new neurons at juvenile stages, but transition to a quiescent state at adulthood with only very rare neurogenesis. Importantly, BrdU pulse chase experiments revealed the existence of a proliferative but otherwise marker-negative cell population replenishing the Neurod-positive progenitor pool, which is never exhausted and maintained even in aged animals. Whether the neurogenic niche can adapt and increase its activity after loss of mature neurons due to injury is currently unknown. For instance, it is unclear if the events in the neurogenic niche of the SAG, which is part of the peripheral nervous system, will be similar or identical to the events in the zebrafish central nervous system. Here, low cycling or even quiescent stem cells undergo reactive proliferation upon injury yielding neural progenitors that differentiate during reactive neurogenesis to replace the damaged cells (Kroehne et al., 2011; Lenkowski and Raymond, 2014). This sequence of events is in stark contrast to the lesion response in the mammalian central nervous system, where reactive proliferation occurs upon lesion, but results in gliosis instead of the production of neural progenitors undergoing reactive neurogenesis (Adams and Gallo, 2018).

In this study, we employed a novel lesion paradigm resulting in a traumatic injury of the adult zebrafish SAG. The mechanical lesion caused an infiltration of blood and immune cells in the lesioned SAG, which persisted beyond 16 days post lesion (dpl). We found that mature SAG neurons underwent apoptosis, which was compensated by increased neurogenesis from the Neurod-positive progenitor pool as early as 4 dpl and showed the highest neurogenesis rate at 8 dpl. Moreover, the Neurod-positive progenitors re-entered the cell cycle displaying, similar to the neurogenesis rate, a peak in proliferation at 8 days post lesion before converging to homeostatic levels at 57 days post lesion. BrdU pulse chase experiments revealed that newborn neurons developed within 14 days after proliferation of the neuronal progenitor cell. In summary, we established a mechanical lesion paradigm of the adult zebrafish SAG eliciting a strong regeneration response characterized by reactive proliferation and reactive neurogenesis. Understanding the cellular and molecular basis of neural regeneration in the adult zebrafish inner ear will reveal differences and similarities to mammalian otic neurogenesis and, thereby, identify zebrafish-specific modules of regeneration as well as mammalian-specific roadblocks.

## Materials and methods

### Ethical Statement

Fish were kept according to FELASA guidelines (Aleström et al., 2020). All animal experiments were conducted according to the guidelines and under supervision of the Regierungspräsidium Dresden (permits: TVV 79/2016, TVA 1/2017 and TVV 21/2018). All efforts were made to minimize animal suffering and the number of animals used.

### Zebrafish Husbandry and Lines

Zebrafish were kept and bred according to standard procedures (Westerfield, 2000; Brand et al., 2002). Our experiments were carried out using adult zebrafish (6 months or older) from wild-type stocks with the AB genetic background and transgenic lines as the following: *TgBAC(neurod1:EGFP)nl1*, abbreviated *neurod*:GFP (Obholzer et al., 2008); *Tg(-3.5ubb:SECHsa.ANXA5-mVenus,cryaa:mCherry)*, abbreviated *ubiq:*secAnnexinV-mVenus (Morsch et al., 2015); and *Tg(elavl3:EGFP)* (Park et al., 2000). Besides date of birth and genotype, also sex and body length measured from the tip of the mouth to the basis of the caudal fin of every analyzed fish were taken.

### Lesion of the Statoacoustic Ganglion *via* Injection Into the Otic Capsule

To study the regenerative capacity of the zebrafish statoacoustic ganglion, we established a lesion assay based on fluid injection into the otic capsule. Fish were anesthetized in Tricaine (Sigma) diluted in fresh fishwater. During the procedure, the anesthetized fish were placed left-sided under a stereomicroscope with dorsal to the top and rostral to the right on a tissue soaked with the Tricaine–fishwater solution laying on a petri dish. Animals were covered with a second Tricaine–fishwater-soaked tissue to keep fish moist. After creating a small hole in the triangular-shaped bone above the gills at the height of the cerebellum using a 20-gauge needle, a Hamilton syringe was carefully inserted approximately 1.2 to 1.5 mm vertically along the dorsal–ventral axis to place it above the anterior part of the SAG without rupturing it. To visualize the area of injection and prove the fluid administration to the SAG, a fluorescent dye (CellTracker™ Red CMTPX Dye, Thermo Fisher Scientific) was slowly injected into the otic capsule in a first round of experimental trials. For all other experiments, three different approaches were performed, namely, sham treatment, NaCl injection, and ouabain injection. For sham-treated animals, the Hamilton syringe was removed from the otic capsule without injecting any fluid. For NaCl and ouabain injections, 0.5–0.8 µl of fluid (0.9% NaCl solution or 100 µM ouabain, dissolved in 0.9% NaCl) was slowly injected into the otic capsule. Afterward, fish were transferred to cages with fresh fishwater for recovery. Around 60%–90% of all treated fish temporarily showed circular swimming behavior after regaining consciousness, which usually lasted for 15–30 min and only in very rare cases longer than 1 h. Minor bleedings at the injected side occurred in 50%–75% of all experimental fish.

### Bromodeoxyuridine Pulse Chase Experiment

To label cells in the S-phase of the cell cycle, transgenic *neurod*:GFP zebrafish were immersed twice in 5 mM bromodeoxyuridine (BrdU) (Sigma) solution (modified after Grandel et al., 2006) for each 24 h from 2 to 3 and from 4 to 5 days post lesion with a 24-h resting period. BrdU was dissolved in E3 medium, and the pH was adjusted to pH 7.2–7.4 using freshly prepared HEPES buffer. Up to five fish were kept under oxygen supply in beakers containing 500 ml of the 5 mM BrdU solution. After the second BrdU pulse, fish were kept in the fish facility for 14 days prior to analysis.

### Tissue Preparation for Immunohistochemistry

Experimental animals were sacrificed, and the skull plate was carefully removed with forceps to enable better fixation of the otic tissue underneath the brain. Samples were fixed overnight in 4% paraformaldehyde in 0.1 M phosphate buffer, pH 7.5 at 4°C, and then incubated minimum overnight in 20% sucrose/20% EDTA in 0.1 M phosphate buffer, pH 7.5 for decalcification and cryoprotection. Subsequently, samples were embedded in 7.5% gelatin/20% sucrose and stored at −80°C. Samples were cut into 12-µm-thick transverse sections and split into two series using a Thermo Scientific CryoStar NX70 Cryostat. Afterward, sectioned samples were stored at −20°C.

### Modifications for CellTracker Red Dye

Samples from CellTracker red dye-injected fish were washed three times in PBS containing 0.3% Triton X-100 (PBSTx 0.3%) for 10 min. Subsequently, samples were incubated with 4,6-diamidino-2-phenylindole (DAPI 1 μg/ml, Invitrogen), diluted in PBSTx 0.3% for 30 min to counterstain the nuclei, washed again two times in PBSTx 0.3% and once in PBS before mounting them in 80% glycerol/PBS.

### Immunohistochemistry

For immunohistochemistry, sections were postfixed with 100% ice-cold methanol at room temperature until all methanol was evaporated (approximately 20–30 min). Afterward, sections were washed once with PBS and three times with PBS containing 0.3% Triton X-100 (PBSTx 0.3%). Incubation with primary antibodies in PBSTx 0.3% (see Table 1) was performed overnight at 4°C in a humid chamber. On the next day, sections were washed three times with PBSTx 0.3% and incubated with Alexa 488-, 555-, 633-, and 647-conjugated secondary antibodies (Thermo Fisher Scientific) in PBSTx 0.3% for 2 h at room temperature. To counterstain the nuclei, 4,6-diamidino-2-phenylindole (DAPI 1 μg/ml, Invitrogen) was added to the secondary antibody-containing solution. Sections were washed again three times with PBSTx 0.3% and once with PBS, and were mounted in 80% glycerol/PBS. Mounted samples were stored in the dark at 4°C.

**TABLE 1:** Primary antibodies used for immunohistochemistry on sections.

Antigen	Species	Isotype	Company	Catalog code	Dilution
Bromodeoxyuridine (BrdU)	Rat	Polyclonal	Abcam	ab6326	1:500
Calretinin	Rabbit	Polyclonal	Swant Inc.	7,699/4	1:2,000
Green Fluorescent Protein (GFP)	Chicken	Polyclonal	Abcam	ab13970	1:2,000
Neural Hu Proteins C and D (HuC/D)	Mouse	IgG2b, k	Thermo Fisher Scientific	A21271	1:500–1:300
Lymphocyte Cytosolic Protein 1 (L-Plastin)	Rabbit	Polyclonal	Generated by the CRTD Antibody Facility		1:1,000
Proliferating Cell Nuclear Antigen (PCNA)	Mouse	IgG2a, k	Dako	M0879	1:500

### Antigen Retrieval for Proliferating Cells in the Area of the Neurogenic Niche and HuC/D Staining

For simultaneous immunohistochemistry against PCNA and HuC/D, an antigen retrieval was performed prior to incubation with primary antibodies. Therefore, slides were incubated in 85°C preheated sodium citrate buffer, pH 6 (10 mM) for 15 min, followed by a 10-min cool down in the heated sodium citrate buffer at room temperature.

### Modifications for Bromodeoxyuridine Staining

Since BrdU and HuC/D require different antigen retrieval methods, immunohistochemistry was performed sequentially. First, samples were stained for *neurod*:GFP and HuC/D as described above without adding DAPI while incubating with the secondary antibodies. Instead of mounting, samples were washed again with PBSTx 0.3%, and afterwards, slides were incubated for 13 min at 37°C in preheated 2 M HCl to retrieve the antigen of BrdU. Subsequently, slides were washed for 10 min in tetraborate buffer and then with PBSTx 0.3%. Slides were incubated with primary antibody against BrdU overnight at 4°C, washed with PBSTx 0.3%, incubated with all secondary antibodies plus DAPI for 2 h at room temperature, and washed again and mounted as described above.

### TUNEL Assay

To analyze the presence of apoptotic cells, the ApopTag Red *In Situ* Apoptosis Detection Kit (ApopTag Chemicon S7165) was used according to the manufacturer’s instructions to perform terminal deoxynucleotidyl transferase-mediated biotinylated UTP nick-end labeling (TUNEL). Since the TUNEL assay was combined with neural staining (*elavl3*:GFP or calretinin), samples were washed three times in PBSTx 0.3% after the rhodamine staining, and subsequent incubation with primary and secondary antibodies was performed as described above.

### Image Acquisition

Images were taken in the light microscopy facility of the CMCB technology platform with a ZEISS Axio Imager, upright microscope with Zeiss Plan-Apochromat 10× 0.45, 20× 0.8, and 40× 0.95 Korr objectives for magnification. An Axiocam MRm (1,388 × 1,040 dexel, 6.45 × 6.45 µm)—b/w was used for detection. Sequential image acquisition was used in samples costained with multiple fluorophores. For imaging, Z-stacks were acquired to cover the full information in all three dimensions (typically, 5–25 Z-planes with an interval of 0.4–1.5 µm depending on the NA of the objective). Images were processed using ZEN blue 2012 and Fiji. Close-up images were generated from the original overview image. For overview images, a maximum projection was taken from several Z-planes, whereas only two to three Z-planes were used for the close-up images. Figures were assembled using Adobe Illustrator 2021.

### Quantification and Statistical Analysis

Quantification was performed on acquired images comprising all Z-planes using the Fiji software. For analysis, the left and right SAG of each fish were taken: the untreated left SAG served as an internal control, whereas the right SAG was treated as described above. To analyze proliferation and neurogenesis upon lesion in the neurogenic niche, a square covering the neurogenic area and the surrounding cells was chosen as the region of interests (see overview images in Figures 4 and 6: size of square: 156.63 mm^2^). From each individual, three sections of one series of the anterior SAG showing the most *neurod*:GFP/PCNA-double positive cells were chosen for quantification. In cases there are less than three sections containing *neurod:*GFP/PCNA-double positive cells in the neurogenic niche, up to three sections with the most *neurod*:GFP-positive cells were chosen. The following cell types were quantified in the region of interest: all *neurod*:GFP-positive cells, *neurod*:GFP/PCNA-double positive cells, *neurod*:GFP/HuC/D-double positive cells, and all PCNA-positive cells. All values from one fish were averaged, and the mean cell count/section was used for statistical analysis (*n* = 3 fish for all conditions and time points; exception: NaCl injected at 4 dpl). For statistical analysis, the graph pad prism software was used to determine *p*-values with a two-way ANOVA test with a Tukey’s multiple comparison test for *post-hoc* analysis. The chosen significance levels are ***p ≤ 0.001, **p ≤ 0.01, *p ≤ 0.05; values above *p* > 0.05 are not considered significant. Graphs are shown as scatter plots with bars with SEM.

## Results

### Establishment of an Injection Procedure to Label the Adult Statoacoustic Ganglion

**FIGURE 1:**
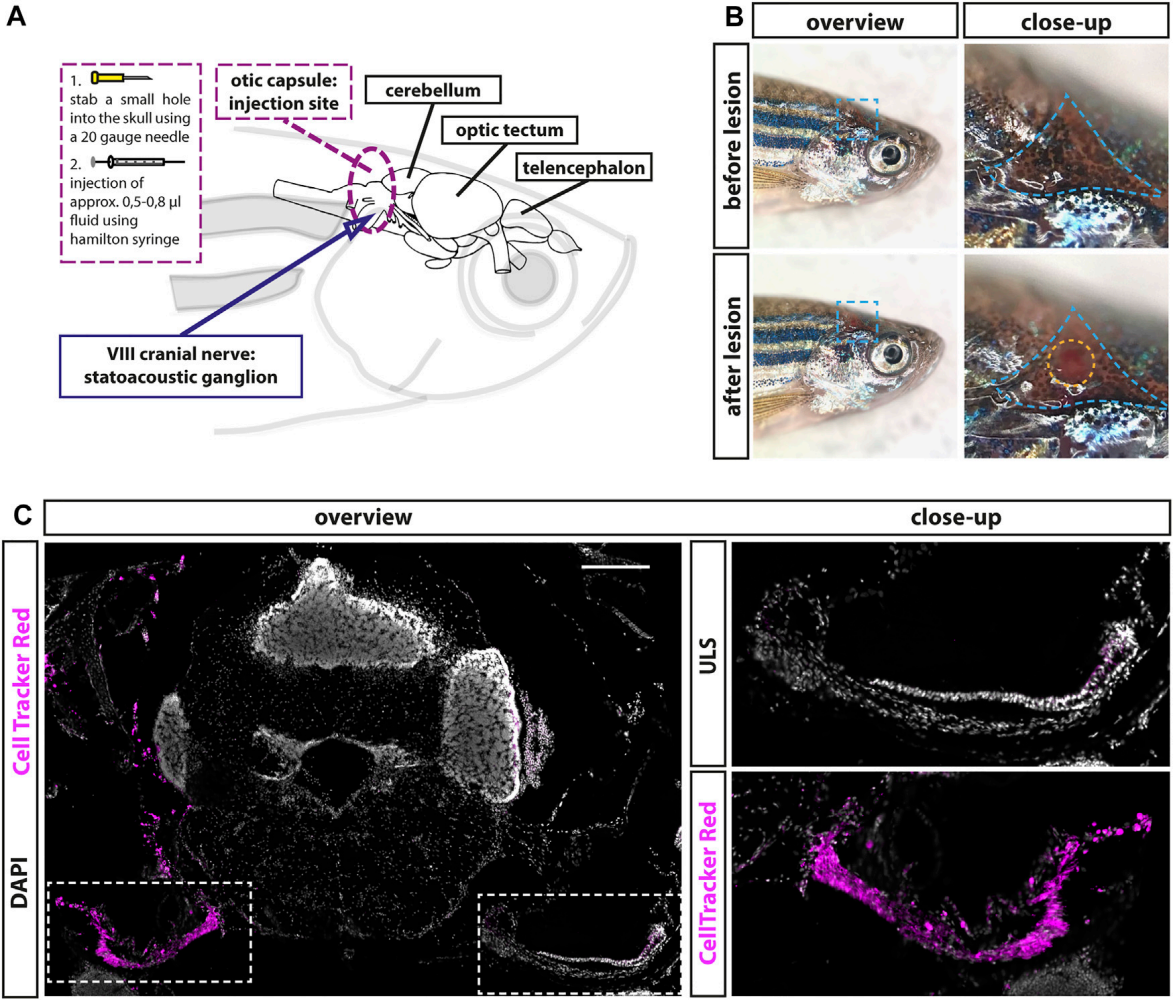
Establishment of a lesion paradigm of the adult zebrafish statoacoustic ganglion (SAG). **(A)** Scheme of the zebrafish head with the brain inside showing the injection position in relation to the brain and the SAG. A 20-gauge needle was used to open the skull before inserting a Hamilton syringe vertically approximately 1.2- to 1.5-mm deep into the skull along the dorsal–ventral axis. Subsequently, the Hamilton syringe was either removed (sham-treated control) or 5–8 µl of either 0.9% NaCl or 100 µM ouabain solution was slowly injected. **(B)** Overview of live adult zebrafish before and after injection. Close-ups of the blue dotted boxes show the injection side within the triangular bone (dotted blue line). Lower close-up shows the hole after injection (dotted yellow circle). **(C)** Immunohistochemistry of a transverse cross section at the height of the anterior SAG following CellTracker Red injection into the right inner ear showed a strong labeling of the SAG on the injected side but no labeling of the unlesioned side (ULS). Scale bar: 200 µm.

**FIGURE 2:**
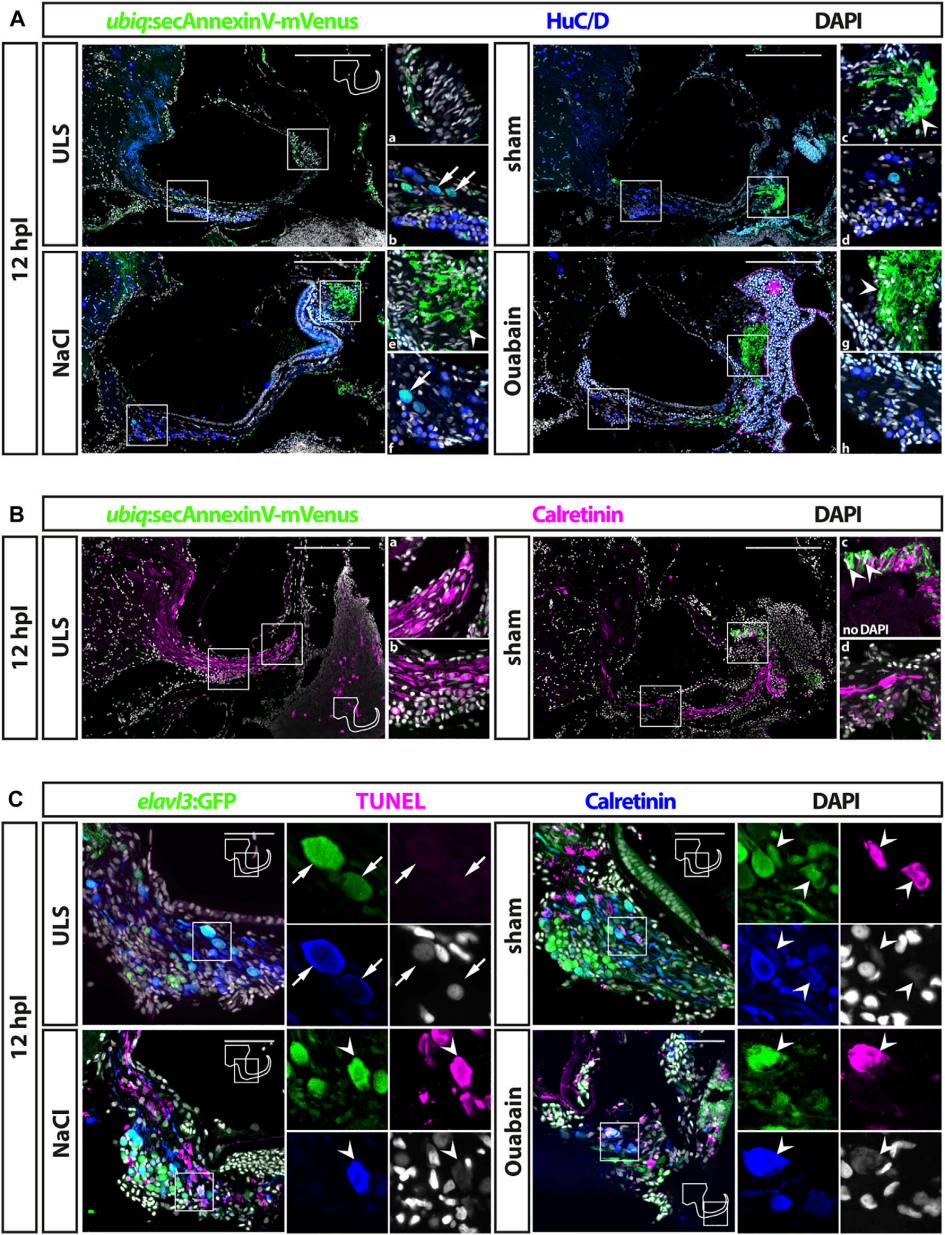
Cell death in the adult zebrafish SAG upon lesion. **(A)** Antibody staining of the adult zebrafish SAG labeling *ubiq:*secAnnexinV-mVenus-positive cells and HuC/D-positive neurons. In contrast to unlesioned sides (ULS), a strong *ubiq:*secAnnexinV-mVenus-signal was present in the SAG following sham treatment or NaCl and Ouabain injections at 12 h post lesion (hpl). In the medial part of the SAG, which harbors the neurogenic niche and the neuronal cell bodies, only very few HuC/D-positive cells showed a weak *ubiq:*secAnnexinV-mVenus staining in all samples (arrows in b and f). In the distal part of the SAG, where the neurites of the sensory neurons innervate the sensory patch, a strong *ubiq:*secAnnexinV-mVenus staining was observed in sham-treated as well as NaCl- and ouabain-injected SAGs, but almost no signal was detected in unlesioned control sides (arrowheads in c, e, and g). Magenta dotted line and asterisk in ouabain-injected SAG indicates a massive hemorrhage in this injected site. **(B)** Colabeling of mVenus with calretinin revealed the presence of *ubiq:*secAnnexinV-mVenus in neurites (arrowhead in c) of sham-treated SAGs but not in unlesioned control sides. **(C)** Antibody staining labeling neurons (*elavl3*:GFP and calretinin) combined with TUNEL assay. TUNEL assay revealed presence of apoptotic neurons in the SAG of adult *elavl3*:GFP zebrafish in all three lesion types at 12 hpl, whereas no TUNEL-positive neurons were found in the unlesioned control side. Scale bars: **(A)** 200 μm; **(B)**: 50 µm.

We recently showed the presence of a lifelong but almost quiescent neurogenic niche in the adult zebrafish statoacoustic ganglion (SAG). It comprises a pool of Neurod-positive neuronal progenitor cells, which are replenished by marker-negative presumptive stem cells (Schwarzer et al., 2020). To investigate whether sensory neurons of the adult SAG can regenerate and whether the neurogenic niche is the source of these newly generated sensory neurons, we aimed to establish the first lesion paradigm of the zebrafish SAG. An established model of auditory neuropathy in mammals is the application of ouabain to the round-window membrane of the cochlea. In the Mongolian gerbil, mice, and rats, this lesion paradigm induces a rapid loss of spiral ganglion neurons while leaving the function of sensory hair cells intact (Schmiedt et al., 2002; Lang et al., 2005; Fu et al., 2012; Wakizono et al., 2021). Therefore, we decided to establish a lesion paradigm based on the application of ouabain to the sensory neurons in the adult SAG. Since fish do not possess an outer and middle ear, application of ouabain or any other solution needs to be administered by entering the inner ear enclosed within the skull. After extensively studying the anatomy and position of the SAG within the zebrafish skull, we identified a position, which allowed the insertion of a Hamilton syringe into the otic capsule without piercing the SAG or the neighboring cerebellum (Figure 1A). For the injection procedure, anesthetized animals were placed on their left side, and the skull was opened by puncturing the triangular-shaped area above the opercle in the area of the pterotic bone using a 20-gauge needle (Figure 1B, blue dotted line). Then a Hamilton syringe was inserted approximately 1.0- to 1.5-mm deep vertically along the dorsal–ventral axis into the otic capsule through the hole in the skull, without damaging the underneath-located SAG (Figure 1B, yellow dotted circle). To test the suitability of the potential SAG lesion paradigm, we performed injections with CellTracker Red. CellTracker Red is a stable, nontoxic dye that freely passes through cell membranes into cells, where it is transformed into a cell membrane-impermeable dye that is brightly fluorescent at a physiological pH. Ninety minutes following CellTracker Red injections, fish were sacrificed, sectioned, and colabeled with DAPI to analyze labeled structures. We find that CellTracker Red was predominantly incorporated into cells within the SAG of the injected side labeling the proximal, medial, and distal parts of the SAG (Figure 1C). Importantly, almost no labeling of the neighboring cerebellum was observed. However, we occasionally noticed a striking accumulation of erythrocytes, easily recognized based on their small and condensed nuclei on the injected site indicating the occurrence of a hemorrhage as a side effect of the injection procedure (Figure 2A, ouabain). Taken together, our injection procedure is suitable to target the SAG specifically and can be used to apply fluids into the adult zebrafish SAG.

### Emergence of Apoptotic Sensory Neurons in the Adult Statoacoustic Ganglion Upon Lesion

Following the successful establishment of the injection procedure, we set out to investigate the efficacy of ouabain-induced lesions. To this aim, 0.5–0.8 µl of a 100 µM ouabain solution was slowly injected into the otic capsule of the right side. As controls, we either injected the same amount of a 0.9% NaCl solution or used sham-treated animals in which the Hamilton syringe was inserted and immediately removed without injecting any fluid. In addition, the SAG of the uninjected side of all experimental animals was used as an unlesioned, internal control. To investigate cell death of sensory neurons in the SAG upon treatment, we took two approaches addressing different biochemical hallmarks of apoptosis: 1) presence of phospholipid phosphatidylserines on the outer plasma membrane of cells and 2) the well-established TUNEL assay. In the first approach, we used the transgenic zebrafish line *ubiq:*secAnnexinV-mVenus, which ubiquitously expresses a secreted Annexin 5 fused to mVenus (Morsch et al., 2015). Annexin 5 binds phospholipid phosphatidylserines with a high affinity. Phospholipid phosphatidylserines are normally distributed asymmetrically and are only present on the inside of the cell membrane. During apoptosis, however, phospholipid phosphatidylserines are redistributed to the outer plasma membrane enabling the detection of apoptotic cells *in vitro* and *in vivo* with Annexin 5 coupled to a fluorescent protein (Morsch et al., 2015; van Genderen et al., 2006; van Ham et al., 2010). Immunohistochemistry against mVenus driven from *ubiq:*secAnnexinV-mVenus combined with the neuronal marker HuC/D detected only low mVenus levels in unlesioned control sides (Figure 2A). In the medial part of the SAG, comprising the neurogenic niche and the neuronal cell bodies, a few HuC/D-positive cells displayed a weak mVenus signal (Figure 2Ab). No mVenus was detectable in the distal part of the unlesioned SAG where the neurites of the sensory neurons innervate the sensory patch (Figure 2Aa). In contrast, a strong mVenus signal was present in sham-treated as well as NaCl- and ouabain-injected SAGs at 12 h post lesion (Figure 2A). Although the number of HuC/D and weak mVenus double-positive cells in the medial part was unchanged (Figures 2Ad, Af, Ah), mVenus levels in the distal part of the SAG were significantly increased in all three lesion conditions (Figures 2Ac, Ae, Ag). Combining immunohistochemistry with mVenus driven in *ubiq:*secAnnexinV-mVenus with an antibody staining against calretinin, which labels a subset of mature neurons including their neurites, corroborated this result (Figure 2B). Again, whereas no difference in mVenus expression was found in the medial part of the SAG (Figures 2Bb, Bd), a strong upregulation of mVenus and colocalization with calretinin was observed in the distal part of the SAG in sham-injected animals in comparison with unlesioned controls (Figures 2Ba, Bc). To validate the presence of apoptotic sensory neurons in contrast to mere axon degeneration, we also applied the well-established TUNEL-assay, which detects nuclear DNA fragmentation, a hallmark of apoptosis. To this aim, we employed the transgenic line *Tg(elavl3:GFP)*, which drives GFP under the promoter of the *elavl3* gene (formerly known as HuC) and, thereby, labeling all neurons in the SAG (Park et al., 2000). In addition to GFP and TUNEL, sections were costained with the neuronal marker calretinin (Figure 2C). In unlesioned control sides, *elavl3*:GFP/calretinin double-positive neurons were always present, but we never detected any TUNEL-positive cell within the SAG. In contrast, robust TUNEL staining was found in sham-treated as well as NaCl- and ouabain-injected SAGs at 12 h post lesion. In addition to other, unidentified TUNEL-positive cells, TUNEL/*elavl3*:GFP/calretinin triple-positive neurons could be found in the lesioned SAG irrespective of the type of lesion. Interestingly, all TUNEL/*elavl3*:GFP/calretinin-triple positive neurons were located on the dorsal side of the SAG close to the sensory patch with a greater distance to the area of the neurogenic niche on the ventral side of the SAG. Taken together, we show that insertion of a Hamilton syringe into the otic capsule induces cell death of sensory neurons in the adult SAG but observe no difference in the apoptotic response following sham treatment, and NaCl and ouabain injections.

### Accumulation of Leukocytes in the Adult Statoacoustic Ganglion Persists Beyond 16 days Post Lesion

**FIGURE 3:**
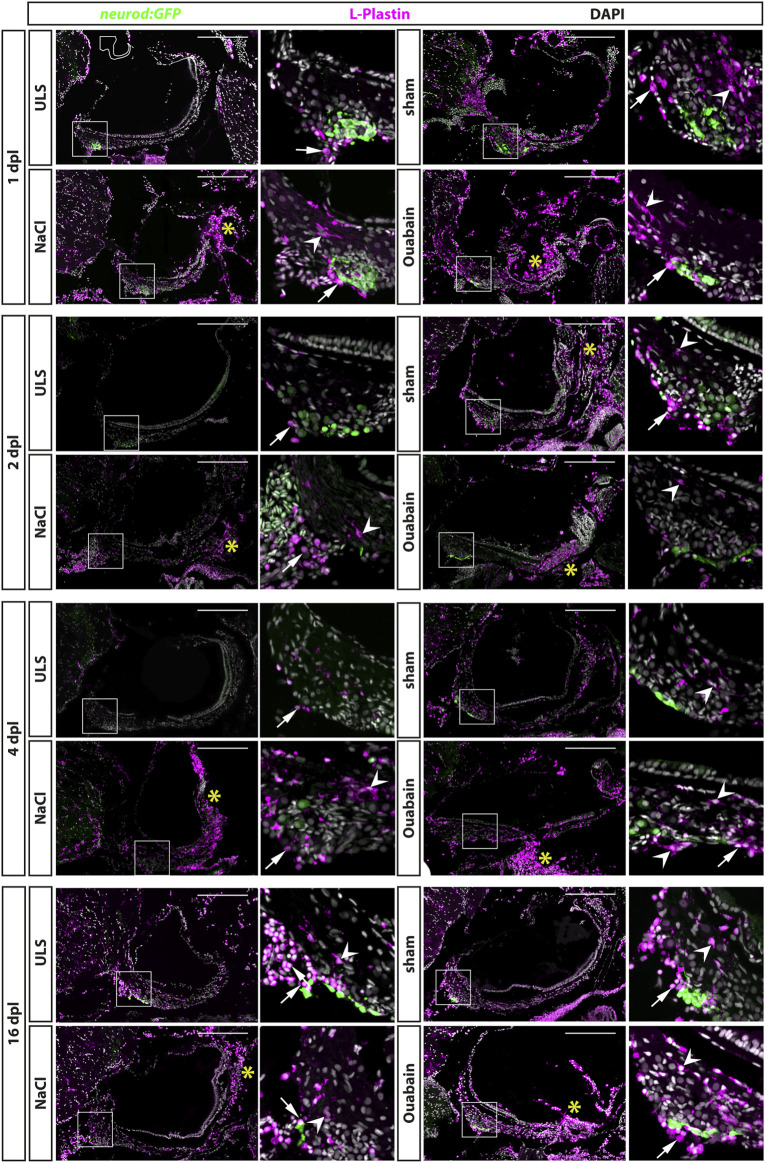
Accumulation of leukocytes in the adult zebrafish SAG upon lesion. Antibody staining against the pan-leukocyte marker L-plastin and GFP in the SAG of transgenic *neurod*:GFP zebrafish at 1, 2, 4, and 16 days post lesion (dpl). Shown are representative images of the SAG following sham-treatment, or NaCl or ouabain injections as well as the unlesioned side (ULS) of either sham-treated, or NaCl- or ouabain-injected animal. Boxes show close-ups of the respective *neurod*:GFP-positive neurogenic niche. In the unlesioned side (ULS, upper left panel in each time point), a few ramified L-plastin-positive cells are distributed evenly throughout the entire SAG. Some round, strong L-plastin-positive cells are localized in close contact to the neurogenic niche on the ventral side of the SAG (arrows). In contrast, accumulation of ramified L-plastin-positive cells (arrowheads) in the SAG is observed close to the neurogenic niche at all time points within sham-treated, or NaCl- or ouabain-injected SAGs (upper right and lower panels in each time point). In addition, the density of L-plastin-positive cells is usually the highest at the distal part of the SAG, especially when the sensory patch is massively destroyed as, for example, seen in 1 dpl/NaCl and ouabain, 2 dpl/ouabain, and 16 dpl/ouabain, irrespective of the lesion type (yellow asterisks). Scale bars: 200 µm.

The immune system plays a crucial but beneficial role in regenerative neurogenesis after injury of the zebrafish central nervous system (Kyritsis et al., 2012). To address whether lesions to the SAG activate the immune system at the lesion side, we performed immunohistochemistry against the pan-leukocyte marker lymphocyte cytosolic protein 1 (L-plastin) on transgenic *neurod*:GFP zebrafish. This way, we were able to label all myelocytes (neutrophils, eosinophils, mast cells, dendritic cells, and macrophages) and lymphocytes (T cells, B cells, and natural killer cells) in relation to the *neurod*:GFP-positive neurogenic progenitor pool of the SAG at 1, 2, 4, and 16 days post lesion (Figure 3). Overall, we distinguished two different types of L-plastin-positive cells: 1) cells with a ramified morphology displaying a weaker L-plastin staining and 2) round cells with a strong L-plastin staining. In unlesioned control sides, we always found only a few, evenly distributed L-plastin-positive cells displaying a ramified morphology within the SAG (Figure 3, ULS in each time point). In addition, round cells with a strong L-plastin staining were located ventrally in close contact to the SAG at the height of the neurogenic niche labeled by *neurod*:GFP. Occasionally, also stronger accumulation of these cells was seen (e.g., Figure 3, ULS at 16 dpl). However, and in contrast, we observed a massive accumulation of ramified as well as round L-plastin-positive cells close to the neurogenic niche and throughout the SAG irrespective of the type of lesion at 1, 2, and 4 dpl. (Figure 3, sham, NaCl, and ouabain in each time point). Moreover, a massive amount of strong L-plastin-positive cells accumulated in the distal part of the SAG where the neurites of the sensory neurons innervate the sensory patch (Figure 3, asterisks). Overall, we found that the accumulation of leukocytes in the SAG is highly similar in sham-treated, and NaCl- or ouabain-injected SAGs. The pattern was already manifested at 1 dpl, did not change much at 2 or 4 dpl, and was even not resolved at 16 dpl, which constituted the end of our time course. Taken together, our data show that sham treatment, or NaCl or ouabain injection into to the SAG results in a massive infiltration and accumulation of immune cells in the lesioned SAG, which lasts longer than 16 dpl.

### Reactive Proliferation in the Neurogenic Niche of the Adult Statoacoustic Ganglion Peaks at 8 days Post Lesion

**FIGURE 4:**
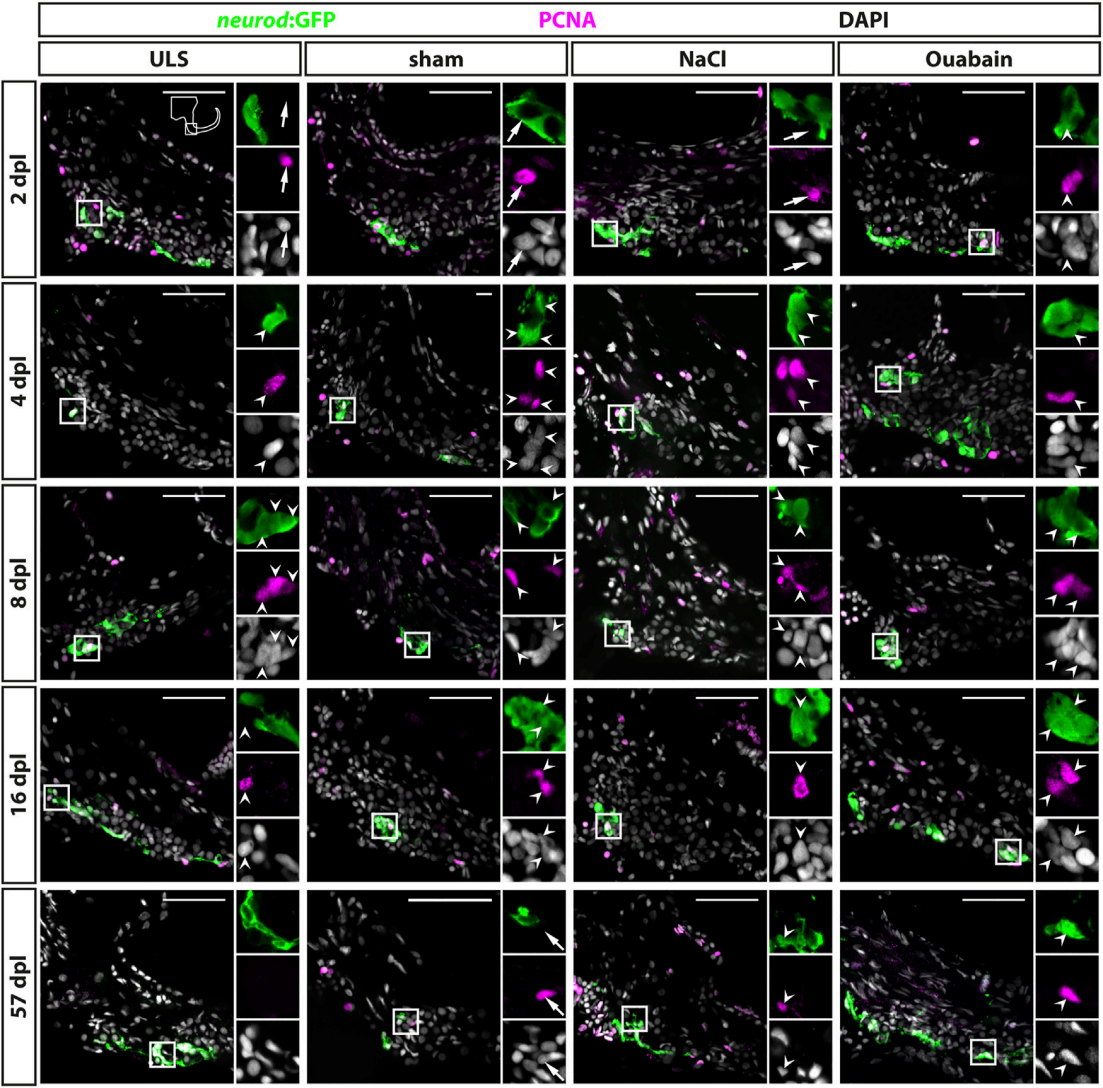
Reactive proliferation of *neurod*:GFP-positive progenitors in the adult zebrafish SAG upon lesion. Immunohistochemistry against the proliferation marker proliferating cell nuclear antigen (PCNA) and GFP in the SAG of *neurod*:GFP transgenic animals. Shown are representative images of the neurogenic niche of the SAG following sham treatment, or NaCl or ouabain injection as well as the unlesioned side (ULS) of either sham-treated, or NaCl- or ouabain-injected animals at 2, 4, 8, 16, and 57 days post lesion (dpl). Boxes show close-ups to visualize costaining. Proliferating but marker-negative cells close to *neurod:*GFP-positive cells (arrows) were found frequently in unlesioned sides, for example, at 2 dpl and in sham-treated SAGs at 57 dpl. However, proliferating *neurod:*GFP/PCNA double-positive cells (arrowheads) were also present from 2 dpl onward in ouabain-injected animals (right column) and frequently from 4 dpl onward in sham-treated and NaCl-injected animals (medial columns). Proliferating *neurod:*GFP/PCNA double-positive cells continued to be present at 8 and 16 dpl before returning to homeostatic levels at 57 dpl when the majority of sham-treated, or NaCl- or ouabain-injected animals did not show proliferating neuronal progenitors in the lesioned SAG. In SAGs of unlesioned sides, PCNA*/neurod*:GFP double-positive cells are found occasionally, in particular, in the unlesioned sides of ouabain-injected fish at 8 dpl. Scale bar: 50 µm.

**FIGURE 5:**
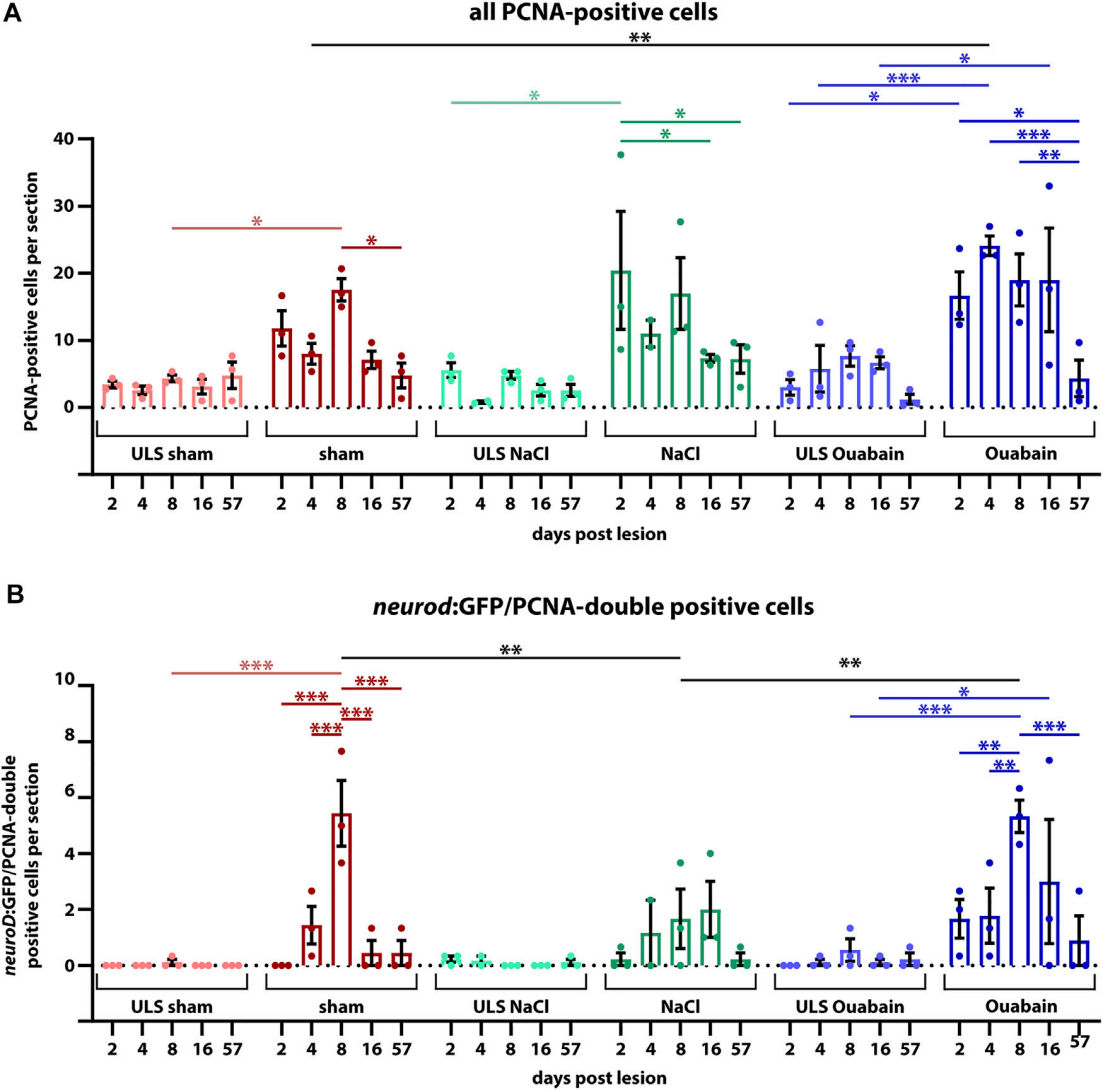
Quantification of reactive proliferation of *neurod*:GFP-positive progenitors. **(A)** Quantification of all PCNA-positive cells. Compared with the respective SAGs of unlesioned sides (ULS sham, ULS NaCl, ULS Ouabain), the number of PCNA-positive cells per section is increased following sham treatment, or NaCl or ouabain injections in the first 8 days post lesion (dpl). An even significant increase compared with the unlesioned side is present at 2 dpl following NaCl injection, at 4 dpl following ouabain injection and at 8 dpl following sham treatment, respectively. At 57 dpl, the proliferation rate in the lesioned SAG was significantly decreased to the levels of the unlesioned side. **(B)** Quantification of *neurod*:GFP/PCNA double-positive cells. Compared with the respective SAGs of unlesioned sides, the number of *neurod*:GFP-positive cells per section that re-enter the cell cycle is increased following sham treatment, or NaCl or ouabain injections. The peak of proliferation of active cycling neuronal progenitors is observed at 8 dpl with a significantly increased number of five to six *neurod*:GFP/PCNA double-positive cells per section in sham-treated and ouabain-injected SAGs. The number of proliferating *neurod*:GFP neuronal progenitors is significantly lower in NaCl-injected SAGs compared with sham-treated and ouabain-injected SAGs at 8 dpl. However, changes in the overall proliferation rate in NaCl-injected SAGs follow a similar trend as seen in sham-treated and ouabain-injected SAGs. The number of *neurod*:GFP/PCNA double-positive cells per section has significantly decreased at 16 and 57 dpl in sham-treated and ouabain-injected SAGs, respectively, although a small number of lesioned animals still showed low numbers of *neurod*:GFP/PCNA double-positive cells in each lesion type. Notably, *neurod*:GFP/PCNA double-positive cells were infrequently also observed in the unlesioned side, in particular, in ouabain-injected animals, in which an average of 0.5–1 *neurod*:GFP/PCNA double-positive cells per section was seen. Samples size *n* = 3 (*n* = fish with three sections/SAG; exception: 4 dpl/NaCl: *n* = 2); data are presented as mean ± SEM. Two-way ANOVA with Tukey’s multiple comparisons test; ***p ≤ 0.001; **p ≤ 0.01; *p ≤ 0.05.

The neurogenic niche in the SAG is highly proliferative and a source of new sensory neurons at juvenile stages but turns to a quiescent state with only very rare neurogenesis at adult stages (Schwarzer et al., 2020). Hence, it was unknown whether the neurogenic niche can be reactivated and provide neuronal cells to compensate for the loss of sensory neurons upon lesion at adult stages. To investigate the regenerative capacity, we used immunohistochemistry against the proliferation marker PCNA and GFP in transgenic *neurod*:GFP zebrafish to check for proliferating cells in the neurogenic niche in sham-treated as well as NaCl- and ouabain-injected animals at 2, 4, 8, 16, and 57 days post lesion (dpl). In contrast to our previous analysis, in which *neurod*:GFP/PCNA double-positive cells were almost always absent in the adult SAG during homeostasis (Schwarzer et al., 2020), we frequently find *neurod*:GFP/PCNA double-positive cells in sham-treated as well as NaCl- and ouabain-injected SAGs (Figure 4). Following injection of ouabain, *neurod*:GFP/PCNA double-positive cells were already present at 2 dpl and were also detected at 4, 8, and 16 dpl (Figure 4, right colomn). Similar results were obtained for sham-treated and NaCl-injected SAGs. Here, *neurod*:GFP/PCNA double-positive cells were frequently detected starting at 4 dpl and were consistently found at 8 and 16 dpl (Figure 4, medial columns). Importantly, *neurod*:GFP/PCNA double-positive cells were also found occasionally in some SAGs of all three lesion types even at 57 dpl, which constituted the end of our time course. This indicates that the neurogenic niche did not return completely to homeostatic levels after lesion at this time point. Surprisingly, *neurod*:GFP/PCNA double-positive cells were also present occasionally in SAGs of the unlesioned side of injected animals (Figure 4, left column). This finding was mostly restricted to 4, 8, and 16 dpl indicating a systemic effect of the unilateral lesion. To analyze the proliferation results in more detail, we quantified the number of all proliferating cells in the area of the neurogenic niche (PCNA-positive) as well as the number of proliferating progenitors (*neurod*:GFP/PCNA double-positive cells). Moreover, instead of combining the results of the unlesioned sides, we kept them individually obtaining the numbers of the unlesioned side for sham-treated, and NaCl- and ouabain-injected, respectively. Quantification of all PCNA-positive cells in the area of the neurogenic niche revealed that the number of proliferating cells in the unlesioned SAG for sham-treated, and NaCl- and ouabain-injected animals does not change significantly over time. It varies to an average of two to eight PCNA-positive cells per section with a slight increased number in unlesioned SAGs of ouabain-injected fish (Figure 5A). Compared with the respective SAGs of unlesioned sides, a significant increase in proliferating cells is observed for sham-treated SAGs at 8 dpl, NaCl-injected at 2 dpl, and ouabain-injected at 4 dpl, respectively. The number of proliferating cells decreased in all three lesion types to an average of six PCNA-positive cells per section within 57 dpl. Comparison of the number of proliferating cells of individual time points from sham-treated, or NaCl- or ouabain-injected SAGs did not reveal any significant differences with one exception. At 4 dpl, the number of PCNA-positive cells was significantly increased in ouabain-injected animals compared with sham-treated animals. Quantification of *neurod*:GFP/PCNA double-positive cells showed that reactive proliferation of neuronal progenitors in the unlesioned side of sham-treated and NaCl-injected animals is a rare event and was observed only once and twice in sham-treated and NaCl-injected animals at 8 and 2 dpl, respectively (Figure 5B). In contrast, one third of all analyzed animals showed low-level reactive proliferation in the neurogenic niche of the unlesioned side following ouabain injection, suggesting that a unilateral ouabain injection influences proliferation in the neurogenic niche of the unlesioned side *via* systemic cues. In the SAG of sham-treated animals, *neurod*:GFP/PCNA double-positive cells are present as early as 4 dpl. The number of proliferating progenitors peaks at 8 dpl, which represents a significant difference in comparison with the unlesioned side, before returning to lower levels at 16 and 57 dpl. Ouabain injection leads to a faster response in the neurogenic niche, where reactive proliferation is already present at 2 dpl. The number of *neurod*:GFP/PCNA double-positive cells increases significantly over the next 6 days and peaks at 8 dpl before it decreases again. A similar profile in the number of proliferating progenitors was observed in the SAGs of NaCl-injected animals, although the overall quantified numbers of *neurod*:GFP/PCNA-proliferating cells was significantly lower at 8 dpl compared with sham-treated and ouabain-injected SAGs. In summary, we show that neuronal progenitors in the neurogenic niche of adult zebrafish SAG indeed re-enter the cell cycle upon lesion and display a peak in reactive proliferation at 8 dpl.

### Reactive Neurogenesis in the Neurogenic Niche of the Adult Statoacoustic Ganglion Peaks at 8 days Post Lesion

**FIGURE 6:**
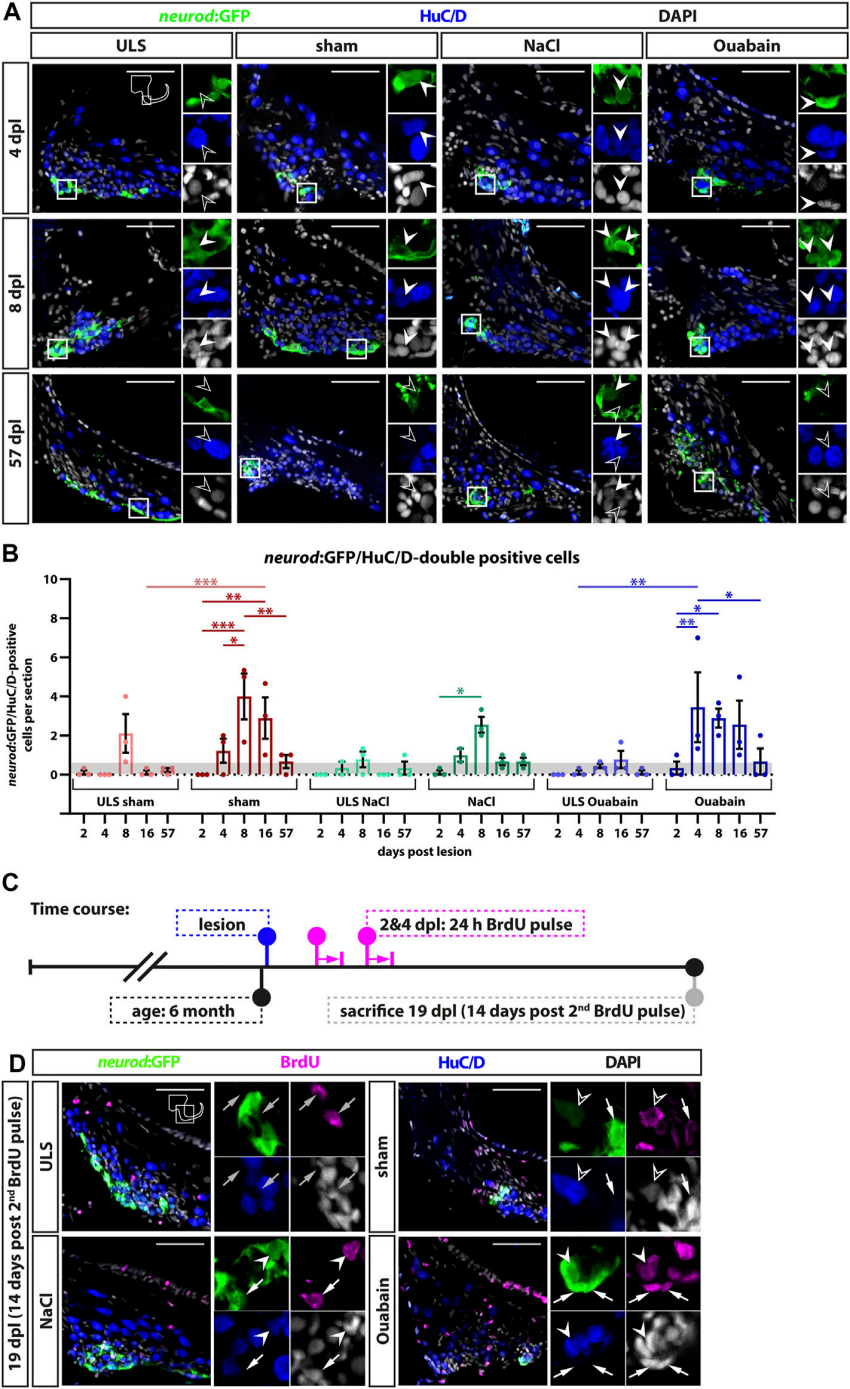
Generation of new sensory neurons from the *neurod*:GFP-positive progenitor pool upon lesion. **(A)** Short-term lineage tracing using the persistence of GFP in the SAG of *neurod*:GFP transgenic animals. Immunohistochemistry against the neuronal marker HuC/D and GFP label newborn neurons in sham-treated, and NaCl- and ouabain-injected animals as well as the unlesioned side (ULS) of either sham-treated, or NaCl- or ouabain-injected animals at 4, 8, and 57 days post lesion (dpl). Shown are representative overviews of the neurogenic niche of the SAG as well as close-ups to visualize co-staining. At 4 dpl, several *neurod*:GFP/HuC/D double-positive cells can be found in the lesioned side independent of the type of lesion, whereas neurogenesis is almost not existing in the corresponding unlesioned side (upper row). At 8 dpl, neurogenesis is abundant in the lesioned sides and increased as well in the respective unlesioned sides in sham-treated, and NaCl- and ouabain-injected animals (middle row). At 57 dpl, neurogenesis returned to low levels in the lesioned and unlesioned side with only a few newborn neurons present in the SAG (lower row). **(B)** Quantification of *neurod*:GFP/HuC/D double-positive cells per section at 2, 4, 8, 16, and 57 days post lesion. The number of *neurod*:GFP/HuC/D double-positive cells per section revealed a significant increase in neurogenesis in the lesioned side in comparison with the homeostatic baseline (0.56 *neurod*:GFP/Huc/D double-positive cells per section) calculated from Schwarzer et al., 2020 (gray box). The number of newborn neurons per section peaked at 4 dpl in ouabain-injected SAGs and at 8 dpl in sham-treated as well as in NaCl-injected SAGs, respectively. At 57 dpl, neurogenesis returned to homeostatic levels in all three lesion conditions. Interestingly, neurogenesis rates in the SAGs of the respective unlesioned sides were also increased over time and slightly above the homeostatic baseline at 8 dpl in sham-treated and NaCl-injected animals as well as at 16 dpl in ouabain-injected animals. Baseline levels in neurogenesis in the respective unlesioned sides was reached again at 16 and 57 dpl, respectively. **(C)** Time course for bromodeoxyuridine (BrdU) pulse chase experiment. Six-month-old *Tg(neurod:GFP)* zebrafish were lesioned, followed by two 24-h lasting BrdU treatments at 2 and 4 days post lesion (dpl). Animals were sacrificed at 19 dpl corresponding to 14 days after the second BrdU treatment. **(D)** Antibody staining against the thymidine analog BrdU, the neuronal marker HuC/D and GFP in the SAG of sham-treated, and NaCl- and ouabain-injected *neurod*:GFP animals at 19 dpl (14 days post second BrdU pulse). In the SAG of the unlesioned side, BrdU-positive cells can be found in close proximity to *neurod*:GFP-positive cells (gray arrow). In contrast, *neurod*:GFP/BrdU double-positive progenitors (arrow), *neurod*:GFP/BrdU/HuC/D triple-positive newborn neurons (arrowhead), and BrdU/HuC/D double-positive neurons (open arrowhead) can be found in the SAG of sham-treated, and NaCl- and ouabain-injected animals. Scale bars: 50 µm. For quantification sample size *n* = 3 (*n* = fish with three sections/SAG; exception: 4 dpl/NaCl: *n* = 2); data are presented as mean ± SEM. Two-way ANOVA with Tukey’s multiple comparisons test; ***p ≤ 0.001; **p ≤ 0.01; *p ≤ 0.05.

During homeostasis, neurogenesis in the zebrafish SAG decreases significantly to very low levels at adult stages (Schwarzer et al, 2020). To investigate whether the adult SAG is capable of reactive neurogenesis upon lesion, we chose two different strategies to prove the presence of newborn neurons: 1) short-term lineage tracing using the persistence of GFP from the *neurod*:GFP transgene in the neuronal progenitors of the neurogenic niche and 2) a pulse chase experiment using bromodeoxyuridine (BrdU). For the former, we employed sham-treated or NaCl-injected or oubain-injected *neurod*:GFP transgenic zebrafish and sacrificed them for analysis at 2, 4, 8, 16, and 57 days post lesion (dpl). Using immunohistochemistry against the neuronal marker HuC/D and GFP, we analyzed the presence of HuC/D-positive neurons carrying the GFP label from the *neurod*:GFP transgene, which represent newborn neurons derived from the *neurod*:GFP-positive neuronal progenitor population. In contrast to the SAG of unlesioned sides, *neurod*:GFP/HuC/D double-positive cells were present in the lesioned SAG irrespective of the type of lesion (sham-treated, NaCl- or ouabain-injected) as early as 4 dpl (Figure 6A). At 8 dpl, *neurod*:GFP/HuC/D double-positive newborn neurons were frequently found in all three lesion types and occasionally in the respective SAGs of the unlesioned sides. At 57 dpl, *neurod*:GFP/HuC/D double-positive cells were rarely found in lesioned as well as in unlesioned SAGs. Quantification of *neurod*:GFP/HuC/D double-positive newborn neurons corroborated a significant increase in neurogenesis upon lesion (Figure 6B). At 2 dpl, the number of *neurod*:GFP/HuC/D double-positive cells per section in unlesioned and lesioned SAGs of all sham-treated, and NaCl- and ouabain-injected animals is less than 0.5, which lies within the range of the homeostatic baseline that we previously described (Schwarzer et al., 2020). However, neurogenesis rates in the SAGs of all three lesion types, but not in the respective SAGs of the unlesioned sides, exceeded the values of the homeostatic baseline already at 4 dpl. The neurogenesis rate increased even further significantly and displayed a peak of three to four *neurod*:GFP/HuC/D double-positive cells per section at 8 dpl in the lesioned SAG after sham treatment, or NaCl or ouabain injection. Interestingly, a slight increase in neurogenesis above the homeostatic baseline was also observed in the unlesioned side of sham-treated and NaCl-injected animals at 8 dpl as well as of ouabain-injected animals at 16 dpl. At 57 dpl, the number of newborn neurons returned to homeostatic levels in lesioned SAGs as well as in the respective unlesioned sides. To analyze whether newborn sensory neurons are derived from progenitors that underwent reactive proliferation, we conducted a BrdU pulse chase experiment. BrdU, a thymidine analog, is incorporated during the S-phase of the cell cycle and, thereby, marks active proliferating cells (Kiernan et al., 2005). To label a great amount of proliferating cells in sham-treated, or NaCl- or ouabain-injected *neurod*:GFP transgenic fish, we immersed lesioned animals from 2 to 3 dpl and from 4 to 5 dpl in a BrdU solution with a 24-h resting period in between (Figure 6C). Our previous BrdU pulse chase experiments of the adult homeostatic zebrafish SAG revealed that BrdU/*neurod*:GFP/HuC/D triple-positive newborn neurons are present at 14 days post-BrdU pulse (Schwarzer et al., 2020). Hence, experimental animals were kept for an additional 14 days, adding up to 19 dpl as the time point of analysis. Antibody staining against BrdU, HuC/D and GFP revealed that BrdU-positive cells are present in the neurogenic niche, but do not colabel with *neurod*:GFP and/or HuC/D in the unlesioned side (Figure 6C). In contrast, we find BrdU/*neurod*:GFP double-positive neuronal progenitors, BrdU/*neurod*:GFP/HuC/D triple-positive newborn neurons, and BrdU/HuC/D double-positive neurons in the lesioned SAG, irrespective of the type of lesion (Figure 6C). In summary, we show that the *neurod*:GFP-positive neuronal progenitor pool acts as a source of newborn neurons also during regeneration and that the neurogenesis rate is indeed transiently and significantly increased upon lesion. Moreover, we find that neurogenesis in the unlesioned side exceeds homeostatic levels, indicating a systemic effect caused by the unilateral lesion.

## Discussion

**FIGURE 7:**
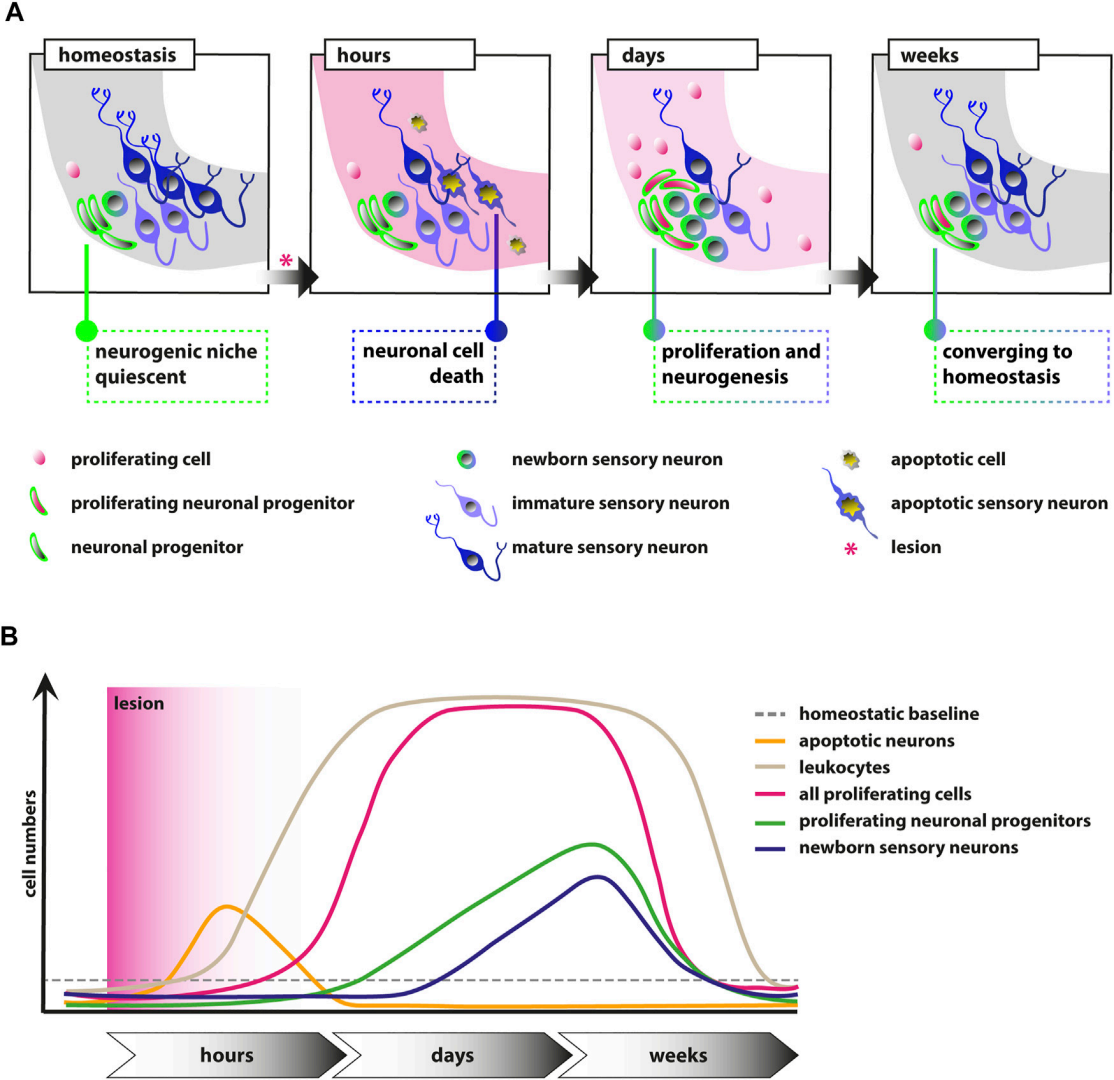
Schematic illustrations of the events in the adult zebrafish SAG upon lesion. **(A)** Scheme depicting the cellular events within the neurogenic niche. During homeostasis, the neurogenic niche of the adult zebrafish SAG is quiescent in regard to proliferation and shows only very rare neurogenesis. Upon lesion, mature sensory neurons as well as other marker-negative cells undergo apoptosis within several hours. Within days, reactive proliferation of marker-negative and neuronal progenitor cells is observed accompanied by reactive neurogenesis from the neuronal progenitor pool. Reactive proliferation and reactive neurogenesis continue over the next weeks until previous homeostatic levels are reached. **(B)** Scheme of the temporal order of events. During homeostasis, only basal levels of proliferating marker-negative cells, proliferating neuronal progenitors, and leukocytes are present in the neurogenic niche of the SAG. In addition, only basal levels of newborn neurons derived from the neuronal progenitors and no apoptotic cells are found. Upon lesion, a strong increase in the number of apoptotic cells and, in particular, apoptotic sensory neurons is observed, which is also resolved rapidly. Also within hours, a massive accumulation of leukocytes occurs, which persists for several weeks. Within days, the number of proliferating cells increases significantly within the neurogenic niche of the SAG and remains high for a couple of weeks until it returns to lower levels. Following the general gain in proliferation, the number of proliferating neuronal progenitors is also significantly increased for approximately 2–3 weeks until it returns to baseline levels. Onset of proliferation of neuronal progenitors is accompanied by increased production of newborn neurons, which remains high for approximately 2–3 weeks until it returns to homeostatic levels.

Understanding the cellular and molecular properties of sensory and neural progenitors are key to develop cell-based therapies using transplantation or reprogramming. To this aim, insights from regeneration-competent as well as regeneration-incompetent organisms complement current *in vitro* approaches. We previously showed that the neurogenic niche of the SAG in the regeneration-competent zebrafish is quiescent with only very rare neurogenesis during homeostasis (Schwarzer et al., 2020). Here, we now show that the neurogenic niche of the eighth cranial nerve in the adult zebrafish displays reactive proliferation as well as reactive neurogenesis in response to a traumatic injury. Upon a traumatic lesion using sham treatment, or NaCl or ouabain injections into the right otic capsule, mature sensory neurons are lost within hours, which is compensated by reactive proliferation of marker-negative and neuronal progenitor cells in the subsequent days and weeks (Figure 7A). In addition, reactive neurogenesis from the neuronal progenitor pool results in the production of newborn sensory neurons. Reactive proliferation and reactive neurogenesis continue over the next weeks until former homeostatic levels are reached. In general, we observed no fundamental differences in the three lesion types except an additional increased level of proliferation in ouabain-injected SAGs in comparison with sham-treated and NaCl-injected SAGs. Hence, we conclude that the insertion of a Hamilton syringe acts as a mechanical lesion and is already sufficient to elicit a regeneration response without any fluid injection. Similar to the regeneration of the zebrafish central nervous system, we find reactive proliferation and reactive neurogenesis, which have been described in detail for the neural retina and forebrain (Kroehne et al., 2011; Lenkowski and Raymond, 2014). Upon lesion of the zebrafish retina, quiescent Müller glia re-enter the cell cycle resulting in the production of multipotent progenitors that differentiate into all retinal cell types replacing the lost cells (Lenkowski and Raymond, 2014). Similarly, radial glia serve as neuronal stem cells in the zebrafish forebrain and show increased production of neuronal progenitors following injury (Kroehne et al., 2011). However, in contrast to the findings in the central nervous system in which reactive proliferation always precedes reactive neurogenesis, we observe, to a large extent, simultaneous reactive proliferation and reactive neurogenesis (Figure 7B). Hence, we hypothesize that the initial reactive neurogenesis is not dependent on previous proliferation of the neuronal progenitor but rather driven by the existing Neurod-positive progenitor pool, which is subsequently replenished by reactive proliferation of Neurod-positive progenitor cells as well as potentially by our previously postulated presumptive marker-negative stem cells. It will now be crucial to identify the nature of the presumptive stem cells and their progenitors as well as the underlying genes and pathways active during homeostasis and regeneration. To this end, single-cell RNA sequencing (scRNAseq) will represent the method of choice. The advent of high-throughput, cost-effective droplet-based scRNAseq allows the assembly of transcriptomic atlases of whole organs including the inner ear (Wu et al., 2021; Yamamoto et al., 2021). Importantly, in addition to scRNAseq data from homeostatic tissues, the transcriptional changes following trauma will be of high importance as recently pioneered by Milon and others (Milon et al., 2021). The comprehensive information gained from these datasets enables insights into the complete cellular composition of the entire tissue and the transcriptomic state of all cell populations within. Moreover, computational algorithms are available that infer a pseudotemporal ordering of cells based on comparative transcriptomic profiling, allowing to derive differentiation trajectories from stem cell or progenitor populations toward differentiated somatic cells in a given tissue or organism (Plass et al., 2018). Once established, interspecies comparisons of scRNAseq datasets from regeneration-competent as well as regeneration-incompetent organisms will identify specific modules of regeneration as well as potential mammalian-specific roadblocks. The employment of recently established conditional gene-targeting approaches, including traditional loxP-flanked alleles or more recent CRISPR/Cas9-based strategies (Hoshijima et al., 2016; Burg et al., 2018; Wu and Wang, 2020; Hans et al., 2021), will enable the rapid genetic analyses of identified pathways and provide important insights also for the currently undertaken *in vitro* approaches.

Although we show that our mechanical lesion paradigm elicits a strong regeneration response within the adult zebrafish SAG, the underlying mechanism remains unclear. The subsequent neuron loss might represent a direct and local effect on the cell cycle status of presumptive stem cells and *neurod*:GFP-positive progenitors. In this respect, it has been reported that dying retinal neurons produce tumor necrosis factor α, a secreted cytokine that is necessary and sufficient for retinal stem cells to initiate cell cycle re-entry during zebrafish retina regeneration (Nelson et al., 2013). Similarly, expression of many other mitogens from local sources has been described to induce proliferation of former quiescent stem cells during regeneration (Fuchs and Steller, 2015). Trauma to the sensory patches after insertion of the Hamilton syringe into the otic capsule, which generates an opening in the usually enclosed inner ear, might provide an additional local cue in our lesion paradigm because strong re-formation of sensory hair cells accompanies neuronal regeneration. However, in addition to local cues, systemic cues must play an important role because we observe reactive proliferation and reactive neurogenesis in the unlesioned side following a unilateral lesion, in particular, following ouabain injections. In this aspect, various systemic effects, including steroids such as estrogen or thyroid hormone, as well as peptide hormones, such as insulin or leptin, have been shown to act as mediators of complex tissue regeneration (Losner et al., 2021). With respect to leptin function, epigenetic profiling provided even evidence for regeneration-dependent enhancer elements in the regeneration-competent zebrafish that are also able to control expression in transgenic mice in a lesion-dependent context (Kang et al., 2016). Another, systemic response that elicits its effect locally constitutes the immune system. In our lesion paradigm, we observe a massive accumulation of leukocytes in the SAG upon injury, which is not resolved at 16 days post lesion, which constituted the end of our time course. In the zebrafish central nervous system, it has been shown that acute inflammation is required for the proper induction of regenerative neurogenesis from neural stem cells (Kyritsis et al., 2012; Tsarouchas et al., 2018; Silva et al., 2020). In particular, the cellular and molecular events during spinal cord regeneration have been dissected in great detail (Tsata and Wehner, 2021). Upon injury, peripheral neutrophils invade the lesion side immediately followed by macrophages, which play a pivotal role in the subsequent functional regeneration, by producing tumor necrosis factor α and controlling interleukin-1 β-mediated inflammation (Tsarouchas et al., 2018). Similarly, T-cells rapidly infiltrate the damaged spinal cord and stimulate regenerative precursor cell proliferation by producing proregenerative factors (Hui et al., 2017). It will be interesting to see if the very same or different mechanisms promote neuronal regeneration in the regenerating adult zebrafish SAG. In this aspect, we would expect several differences because in contrast to the central nervous system in which the number of infiltrated immune cells following injury is decreased within days (Kyritsis et al., 2012), we see persistent presence of immune cells in the lesioned SAG.

Our novel lesion paradigm shows that the neurogenic niche within the adult zebrafish SAG displays a strong regeneration response upon injury, which provides us a first entry point to address the underlying cellular and molecular events. However, to in-depthly understand the regeneration of the SAG, the establishment of various alternative lesion paradigms would be beneficial. For instance, in addition to mechanical lesions, several other injury models are available to address regeneration of the adult retina, including intense light, application of neurotoxins, as well as pharmacogenetic approaches (Fimbel et al., 2007; Montgomery et al., 2010; Weber et al., 2013). In particular, the latter provides a conditional and unique platform for the precise ablation of cells in a tissue- or cell type-specific manner. Commonly, the nitroreductase/metronidazole system is used in which the bacterial nitroreductase expressed by a tissue- or cell type-specific transgene converts the prodrug metronidazole into a cytotoxic DNA cross-linking agent (Curado et al., 2007). Similarly, when using the human diphtheria toxin receptor/diphtheria toxin system, cells specifically expressing the human diphtheria toxin receptor undergo apoptosis after application of diphtheria toxin (Jimenez et al., 2021). Application of these alternative lesion paradigms or even their combinations will help to dissect the mechanistic underpinnings of successful SAG regeneration.

## Data Availability

The original contributions presented in the study are included in the article/Supplementary Material. Further inquiries can be directed to the corresponding author.
